# Epidemiology of the association between serum 25-hydroxyvitamin D levels and musculoskeletal conditions among elderly individuals: a literature review

**DOI:** 10.1186/s40101-020-00249-3

**Published:** 2020-11-26

**Authors:** Kazuhiko Arima, Satoshi Mizukami, Takayuki Nishimura, Yoshihito Tomita, Hiroki Nakashima, Yasuyo Abe, Kiyoshi Aoyagi

**Affiliations:** 1 Department of Public Health, Nagasaki University Graduate School of Biomedical Sciences, Nagasaki, Japan; 2 Department of Human Science, Faculty of Design, Kyushu University, Fukuoka, Japan; 3 School of Rehabilitation, Department of Physical Therapy, Tokyo Professional University of Health Sciences, Tokyo, Japan

## Abstract

**Background:**

Vitamin D deficiency is associated with osteoporosis, fracture, muscle weakness, falls, and osteoarthritis in adults. Elderly individuals are more likely to present with poor musculoskeletal conditions. Recently, several epidemiological studies have assessed the correlation between serum 25-hydroxyvitamin D (25(OH)D) levels and musculoskeletal conditions in elderly individuals.

**Main text:**

Osteoporosis is a skeletal disease characterized by low bone mass and microarchitectural deterioration of bone tissue, with a consequent increase in bone fragility and susceptibility to fracture. Numerous studies have shown a positive association between serum 25(OH)D levels and bone mineral density. Only a few studies have reported an association between serum 25(OH)D levels and quantitative ultrasound (QUS) parameters. Low serum 25(OH)D level may be a risk factor for hip fracture. However, data on the association between vitamin D deficiency and the incidence of non-hip fracture are contrasting. Falls are a major cause of mortality and morbidity in older adults. Several prospective population-based cohort studies have shown that low 25(OH)D levels are associated with an increased risk of falls. Reduced muscle strength and physical performance are risk factors for adverse events, including disability, institutionalization, and mortality. The role of vitamin D in musculoskeletal functionality (muscle weakness and physical performance) among elderly individuals is still controversial. Osteoarthritis (OA) is the most common form of arthritis and is a leading cause of disability among older adults. Data on the association between serum 25(OH)D levels and OA are contrasting.

**Conclusion:**

Some studies have shown that vitamin D deficiency may be a risk factor for poor musculoskeletal conditions, such as osteoporosis, fracture, muscle weakness, falls, and osteoarthritis in adults. However, other studies did not find an association between serum 25(OH)D levels and musculoskeletal conditions.

## Introduction

Vitamin D is produced in the skin via exposure to ultraviolet (UV) light or intake of food. Then, it is hydroxylated by the liver to 25-hydroxyvitamin D (25(OH)D), which is the primary circulating form, and further hydroxylated by the kidney to 1,25-dihydroxyvitamin D (1,25(OH)_2_D), which is the active form [1]. Thus, environment (exposure to UV radiation from the sun) and nutrition (intake of vitamin D-rich food or supplements) contribute to vitamin D production.

Vitamin D deficiency is associated with osteoporosis, fracture, muscle weakness, falls, or osteoarthritis in adults [2]. Elderly individuals are more likely to present with poor musculoskeletal conditions. Fractures lead to considerable disability and premature death among elderly individuals [3]. With increased life expectancy, elderly people comprise a high portion of the community. Deterioration of musculoskeletal health is a serious problem among elderly individuals.

Understanding the relationship among environment, nutrition, and physical (musculoskeletal) conditions is important in physiological anthropology [4–7]. Recently, several epidemiological studies have evaluated the correlation between serum 25(OH)D levels and musculoskeletal conditions among elderly individuals. However, the associations between serum 25(OH)D levels and musculoskeletal conditions remain controversial since previous studies have shown conflicting results. The current study aimed to review the epidemiological topic of serum 25(OH)D levels and its musculoskeletal impacts.

## Osteoporosis

Osteoporosis is a skeletal disease characterized by low bone mass and microarchitectural deterioration of bone tissue with a consequent increase in bone fragility and susceptibility to fracture [8].

### Bone mass: bone mineral density (BMD) and quantitative ultrasound (QUS) parameters

The measurement of bone mass (BMD) is the most straightforward approach for the diagnosis of osteoporosis [9]. Several studies have shown a positive association between serum 25(OH)D levels and BMD.

Bischoff-Ferrari et al. [10] showed a significant positive association between serum 25(OH)D levels and total hip BMD in older white, Mexican American, and black adults in the USA. Nakamura et al. [11] have found that higher serum 25(OH)D concentrations are associated with increased femoral neck BMD in home-dwelling postmenopausal Japanese women. Moreover, a serum 25(OH)D concentration of at least 70 nmol/L is required to achieve a high femoral neck BMD, and at least 50 nmol/L is needed to achieve normal parathyroid hormone levels and to prevent low BMD. Mezquita-Raya et al. [12] have found that lumbar spine BMD is significantly associated with 25(OH)D levels in 161 healthy postmenopausal women. Malavolta et al. [13] have revealed a significant positive correlation between spine and hip BMD and 25(OH)D levels in 156 Italian postmenopausal women. Yamauchi et al. [14] showed that BMD was significantly correlated with 25(OH)D levels after adjusting for age, body mass index, and serum creatinine and parathyroid hormone levels in 202 Japanese postmenopausal women. Liu et al. [15] assessed 4595 participants (2281 men and 2314 women) aged ≥ 50 years who participated in the National Health and Nutrition Examination Survey 2001–2006. Results showed that serum 25(OH)D levels were positively correlated with lumbar BMD in older adults.

The QUS of the bone is an ionizing radiation-free and relatively inexpensive, portable screening technique for osteoporosis and/or fracture risk assessment [16]. A few studies have reported an association between serum 25(OH)D levels and QUS parameters [17, 18]. Two studies found a positive association between serum 25(OH)D levels and speed of sound (SOS) [17, 18]. However, only one study revealed a positive association between serum 25(OH)D levels and broadband ultrasound attenuation (BUA) [17].

Kauppi et al. [17] investigated 2736 men and 3299 women aged ≥ 30 years from a nationally representative population sample in Finland. Results showed that serum 25(OH)D level is an independent determinant of BUA (*p* < 0.0001 for men, *p* < 0.001 for women) and SOS (*p* < 0.0001 for men, *p* < 0.05 for women). Grigoriou et al. [18] investigated 970 adults recruited from rural and urban areas in Greece. Results showed that individuals with 25(OH)D levels ≥ 20 ng/mL had a higher SOS than those with 25(OH)D levels < 20 ng/mL.

### Hip and other types of fracture

Fragility fractures commonly occur among patients with osteoporosis, and they are considered an important public health issue. Low serum 25(OH)D level was found to be a risk factor for hip fracture [19–21].

Looker and Mussolino [19] investigated 1917 white men and women aged ≥ 65 years who participated in the third National Health and Nutrition Examination Survey 1988–1994. They found that serum 25(OH)D level was correlated with a significantly lower risk of hip fracture after adjusting for several relevant confounding variables. Robinson-Cohen et al. [20] assessed 2294 ambulatory older adults (mean age 74 years) in a study with a median follow-up of 13 years. Results showed that serum 25(OH)D concentrations were associated with the risk of long-term hip fracture. Steingrimsdottir et al. [21] evaluated 5764 men and women aged 66–96 years in a study with a mean follow-up period of 5.4 years. They showed that compared with a reference value of 50–75 nmol/L, the hazard ratios for hip fractures were 2.24 (95% confidence interval [CI] 1.63, 3.09) for serum 25(OH)D levels < 30 nmol/L after adjusting for age, sex, body mass index, height, smoking, alcohol intake and season, and 2.08 (95% CI 1.51, 2.87) after adjusting additionally for physical activity.

Data on the association between vitamin D deficiency and the incidence of non-hip fracture are contrasting.

Some studies have reported an association between vitamin D deficiency and the risk of non-hip fracture [22–25]. Bleicher et al. [22] investigated a cohort of 1662 community-dwelling men aged 70–97 years in a study with a mean follow-up period of 4.3 years. They found that the risk of fracture was highest in men with 25(OH)D levels in the lowest quintile (25(OH)D level ≤ 36 nmol/L; hazard ratio [HR] = 3.5; 95% CI 1.7–7.0) and in men with 25(OH)D levels in the highest quintile (25(OH)D level > 72 nmol/L; HR = 2.7; 95% CI 1.4–5.4) compared with men with 25(OH)D levels in the 4th quintile (25(OH)D level ≥ 60 to ≤ 72 nmol/L). This result indicated that extremely high or low serum 25(OH)D levels may have negative effects on fracture risk. Tanaka et al. [23] investigated 1470 postmenopausal Japanese women in a study with a mean follow-up period of 7.2 years. They found that 25(OH)D level was a leading risk factor for long bone fractures. Nakamura et al. [24] conducted a cohort study with a 6-year follow-up on 773 community-dwelling women aged ≥ 69 years old. Results showed that a sufficient vitamin D status (serum 25(OH)D levels ≥ 71 nmol/L) was associated with low limb and vertebral fracture risk. Van Schoor et al. [25] conducted a 6-year follow-up study on 1311 community-dwelling older men and women from the Longitudinal Aging Study Amsterdam. They found that serum 25(OH)D levels ≤ 12 ng/mL were associated with an increased risk of fracture in individuals aged 65–75 years.

Other studies did not reveal any associations between low serum 25(OH)D levels and a higher risk of fracture [26, 27]. Garnero et al. [26] investigated 669 postmenopausal women (mean age 62.2 years) in a prospective study with a mean follow-up period of 11.2 years. Results showed that after adjusting for age, there was no significant difference in incidence of fracture between women with 25(OH)D levels below or above 75, 50, or 30 nmol/L. Chan et al. [27] investigated 712 men aged ≥ 65 years in a 4-year follow-up study. They found no association between serum 25(OH)D levels and risk of non-vertebral fracture or hip fracture after adjusting for confounding factors.

## Falls

Falls are one of the major causes of mortality and morbidity in older adults [28, 29]. The ability of balance and gait control, and musculoskeletal functions are important risk factors for falls [28, 30].

In a cross-sectional study, Suzuki et al. [31] assessed 2957 Japanese community-dwelling elderly individuals (950 men and 2007 women) aged 65–92 years. Results showed that low 25(OH)D levels were significantly associated with a high prevalence of falls in elderly Japanese women.

Several prospective population-based cohort studies have shown that low 25(OH)D levels are associated with an increased risk of falls [32–34]. Rothenbacher et al. [32] evaluated 1385 participants aged ≥ 65 years (mean age 75.6 years) in a 1-year follow-up study. They showed an association between serum 25(OH)D levels and risk of first fall, which was evident in participants with serum calcium levels within the upper normal limit, independent of renal function. Shimizu et al. [33] investigated community-dwelling elderly women aged ≥ 75 years (*n* = 1393) in a 1-year follow-up study. They found that a lower serum 25(OH)D level (< 20 ng/mL) was significantly associated with an increased risk of falls. Machado et al. [34] evaluated 705 community-dwelling elderly individuals (448 women, 257 men) in a study with a mean follow-up period of 4.3 ± 0.8 years. They found that hypovitaminosis D was associated with recurrent falls.

## Muscle weakness and physical performance

Reduced muscle strength and physical performance are risk factors for adverse events, including disability, institutionalization, and mortality [35]. The role of vitamin D in musculoskeletal functionality (muscle weakness and physical performance) among elderly individuals remains controversial.

Some studies have shown an association between vitamin D deficiency and muscle weakness or poor physical performance [36–39]. Bischoff-Ferrari et al. [36] found that in both active and inactive ambulatory persons aged ≥ 60 years, 25(OH)D levels between 40 and 94 nmol/L are associated with better musculoskeletal function in the lower extremities than are levels < 40 nmol/L. Houston et al. [37] conducted a study on 976 individuals aged ≥ 65 years. Results showed that vitamin D levels were significantly associated with Short Physical Performance Battery (SPPB) (walking speed, ability to stand from a chair, and ability to maintain balance in progressively more challenging positions [side-by-side position, semi-tandem position, and full-tandem position]) in men (*p* = 0.04) and handgrip strength in men (*p* = 0.004) and women (*p* = 0.01). Aspell et al. [38] evaluated for handgrip strength and SPPB in 4157 community-dwelling adults with a mean age of 69.8 ± 6.9 years. Their study showed that vitamin D deficiency (< 30 nmol/L) was a significant determinant of low handgrip strength (odds ratio [OR] 1.44 [1.22, 1.71], *p* < 0.001) and poor physical performance (OR 1.65 [1.31, 2.09], *p* < 0.001). Toffanello et al. [39] found that lower 25(OH)D levels were associated with worse coordination and weaker strength (5 timed chair stands) in women, slower walking time, and lower upper limb (handgrip) strength in men and a weaker aerobic capacity (6-min walking) in both genders in 2694 community-dwelling elderly women and men from the Progetto Veneto Anziani study.

Other studies did not show any associations between vitamin D deficiency and muscle weakness or poor physical performance [40–42]. Vaes et al. [40] investigated 756 men and women aged ≥ 65 years. Their study showed that serum 25(OH)D concentrations were significantly associated with frailty status and measures of physical performance, including gait speed and the Timed Up and Go test, but not with strength-related outcomes (handgrip strength or knee-extension strength). Annweiler et al. [41] evaluated the maximal isometric voluntary contraction strength of the lower limb and hand with computerized dynamometers in a randomized sample of 440 women included in the EPIDOS study. Results showed that the mean muscle strength did not differ among the three groups of women (serum 25(OH)D levels < 15 ng/mL, 15–30 ng/mL, and > 30 ng/mL with *p* = 0.946 for the quadriceps and *p* = 0.064 for handgrip). This result does not support the hypothesis stating that a relationship exists between low serum 25(OH)D concentrations and low muscle strength. Mathei et al. [42] found no significant relationship between balance, gait speed, and grip strength as well as serum 25(OH)D levels in 367 individuals aged ≥ 80 years.

## Osteoarthritis

Osteoarthritis (OA) is the most common form of arthritis, affecting approximately 302 million people worldwide. Moreover, it is a leading cause of disability among older adults [43]. Data on the association between serum 25(OH)D levels and OA are contrasting.

Several studies showed the association between serum 25(OH)D level and OA [44–46]. In a cross-sectional study, Veronese et al. [44] found that low 25(OH)D levels were associated with the presence of OA and with OA-related pain, particularly when the hand and hip are involved in 2756 subjects (1102 men and 1654 women) with a mean age of 74.2 ± 7.1 years. Heidari et al. [45] investigated 148 patients with knee OA and 150 controls and found a significant association between serum 25(OH)D deficiency and knee OA in patients aged < 60 years. Zhang et al. [46] evaluated 418 participants who had ≥ 1 knee with both symptomatic and radiographic OA prospectively (between the 24 and 48 months). Results showed that participants with low 25(OH)D levels (< 15 μg/L) had a > 2-fold elevated risk of knee OA progression compared with those with greater vitamin D concentrations (≥ 15 μg/L).

However, other studies did not show an association between low 25(OH)D levels and a higher risk of knee OA [47, 48]. In a cross-sectional study, Muraki et al. [47] found that 25(OH)D levels were not significantly associated with radiographic knee OA in 787 participants in the Hertfordshire Cohort Study (399 men, 388 women; mean age of 65.6 ± 2.7 years). Konstari et al. [48] conducted a cohort study comprising 5274 Finns who participated in a national health examination survey and who had no knee or hip OA at baseline and at the 10-year follow-up. Results showed that a low serum 25(OH)D concentration was not effective in predicting the high incidence of knee and hip OA.

## Conclusion

Some studies showed that vitamin D deficiency may be a risk factor for poor musculoskeletal conditions, such as osteoporosis, fracture, muscle weakness, falls, and osteoarthritis in adults. However, other studies did not show an association between serum 25(OH)D levels and musculoskeletal conditions. Thus, the associations between serum 25(OH)D levels and musculoskeletal conditions were conflicting. Further epidemiological studies should be conducted to explore the association between serum 25(OH)D levels and musculoskeletal conditions. In addition, a systematic review and meta-analysis of randomized trials showed the small benefit of vitamin D supplements at the femoral neck bone mineral density, but not at any other site [49]. Intervention studies on vitamin D supplementation would be needed to clarify the effect of vitamin D on musculoskeletal conditions and its optimal serum levels to maintain a good physical function, thereby improving the health management of elderly individuals.

## Data Availability

Not applicable.

## Abbreviations

UV: Ultraviolet
25(OH)D: 25-Hydroxyvitamin D
1,25(OH)2D: 1,25-Dihydroxyvitamin D
BMD: Bone mineral density
QUS: Quantitative ultrasound
SOS: Speed of sound
BUA: Broadband ultrasound attenuation
SPPB: Short physical performance battery
OA: Osteoarthritis
